# Fetal heart rate evolution patterns in cerebral palsy associated with umbilical cord complications: a nationwide study

**DOI:** 10.1186/s12884-022-04508-2

**Published:** 2022-03-03

**Authors:** Junichi Hasegawa, Masahiro Nakao, Tomoaki Ikeda, Satoshi Toyokawa, Emi Jojima, Shoji Satoh, Kiyotake Ichizuka, Nanako Tamiya, Akihito Nakai, Keiya Fujimori, Tsugio Maeda, Satoru Takeda, Hideaki Suzuki, Shigeru Ueda, Mitsutoshi Iwashita, Tsuyomu Ikenoue

**Affiliations:** 1 Visiting Researcher, Department of the Japan Obstetric Compensation System for Cerebral Palsy in Public Interest Incorporated Foundation, Japan Council for Quality Health Care, Tokyo, Japan; 2 Department of Obstetrics and Gynecology, St. Marianna University School of Medicine, 2-16-1 Sugao, Miyamae-ku, Kawasaki, Kanagawa 216-8511 Japan; 3 Department of Obstetrics and Gynecology, Mie University Graduate School of Medicine, Mie, Japan; 4 Department of Obstetrics and Gynecology, Sakakibara Heart Institute, Tokyo, Japan; 5 The Prevention Recurrence Committee, Japan Obstetric Compensation System for Cerebral Palsy, Tokyo, Japan; 6 Department of Public Health, The University of Tokyo, Tokyo, Japan; 7 Department of the Japan Obstetric Compensation System for Cerebral Palsy, Japan Council for Quality Health Care, Tokyo, Japan; 8 Maternal and Perinatal Care Center, Oita Prefectural Hospital, Oita, Japan; 9 Department of Obstetrics and Gynecology, Showa University Northern Yokohama Hospital, Kanagawa, Japan; 10 Department of Health Services Research, Faculty of Medicine, University of Tsukuba, Ibaraki, Japan; 11 Department of Obstetrics and Gynecology, Nippon Medical School, Tokyo, Japan; 12 Department of Obstetrics and Gynecology, Fukushima Medical University, Fukushima, Japan; 13 Maeda Clinic, Incorporated Association Anzu-kai, Shizuoka, Japan; 14 Department of Obstetrics and Gynecology, Juntendo University Faculty of Medicine, Tokyo, Japan; 15 Kugayama Hospital, Tokyo, Japan; 16 Miyazaki University, Miyazaki, Japan

## Abstract

**Background:**

The aim of the present study was to clarify fetal heart rate (FHR) evolution patterns in infants with cerebral palsy (CP) according to different types of umbilical cord complications.

**Methods:**

This case–control study included children born: with a birth weight   ≥2000 g, at gestational age   ≥33 weeks, with disability due to CP, and between 2009 and 2014. Obstetric characteristics and FHR patterns were compared among patients with CP associated with (126 cases) and without (594 controls) umbilical cord complications.

**Results:**

There were 32 umbilical cord prolapse cases and 94 cases with coexistent antenatal umbilical cord complications. Compared with the control group, the persistent non-reassuring pattern was more frequent in cases with coexistent antenatal umbilical cord complications (*p* = 0.012). A reassuring FHR pattern was observed on admission, but resulted in prolonged deceleration, especially during the first stage of labor, and was significantly identified in 69% of cases with umbilical cord prolapse and 35% of cases with antenatal cord complications, compared to 17% of control cases (*p* < 0.001).

**Conclusion:**

Hypercoiled cord and abnormal placental umbilical cord insertion, may be associated with CP due to acute hypoxic-ischemic injury as well as sub-acute or chronic adverse events during pregnancy, while umbilical cord prolapse may be characterized by acute hypoxic-ischemic injury during delivery.

**Supplementary Information:**

The online version contains supplementary material available at 10.1186/s12884-022-04508-2.

## Background

Hypoxia–ischemia and acidemia are known causes of cerebral palsy (CP), although the etiology of many CP cases remains unknown [1]. Since placental and umbilical cord abnormalities are likely to induce a hypoxic fetal condition, an association between CP and these abnormalities has been previously reported [2, 3]. Our previous nationwide report [4] demonstrated that the major obstetric factors associated with CP after 33 weeks of gestation were placental abnormalities (31%) and umbilical cord abnormalities (15%).

Population-based studies have found that intrapartum hypoxia–ischemia is present in a smaller percentage of term children, with CP ranging from 8 to 28% in developed countries [1, 5, 6]; this rate has persisted over the past 30 years [1]. Therefore, CP may be associated with acute umbilical cord compression as well as chronic insufficient fetoplacental perfusion during the antepartum period. The pathology of umbilical cord abnormalities during labor might be associated with cord vulnerability to external forces during uterine contractions, resulting in acute hypoxia–ischemia (intrapartum hypoxia–ischemia). However, among the types of umbilical cord abnormalities, we hypothesized that an insufficiency in umbilical cord blood flow might vary from the antepartum period to delivery.

Classification of the fetal heart rate (FHR) evolution pattern in CP cases, as described by Phelan et al., is useful for determining the timing of asphyxial events that can cause fetal brain injury [7]. They analyzed intrapartum FHR in brain-damaged term infants, and divided them into the following groups: (1) bradycardia that indicates terminal fetal bradycardia on admission test, (2) a persistent non-reassuring pattern that continues from admission until delivery, (3) reassuring-prolonged deceleration that indicates reassuring admission tests followed by abrupt FHR changes with prolonged deceleration or bradycardia, (4) Hon’s pattern, described as a “stair-step to death” by Edward Hon in 1968, which indicates reassuring admission test findings and recurrent decelerations in FHR, high baseline FHR, and decreased variability followed by low baseline FHR, and (5) a persistent reassuring pattern throughout delivery.

Therefore, we hypothesized that the assessment of FHR evolution patterns would provide clues to the timing and preventability of injury in infants with CP associated with umbilical cord complications. In the present study, we aimed to clarify the FHR evolution patterns in patients with CP according to the different specific types of umbilical cord complications.

## Methods

A case–control study was performed in children with CP who were approved for compensation by a review of the Japan Obstetric Compensation System for Cerebral Palsy (JOCSC). Disability was certified as first- or second-degree severity according to the grade of disability definitions in the Act on Welfare of Physically Disabled Persons.

Among the children born between January 2009 and July 2014, those with a birth weight ≥ 2000 g, gestational age ≥ 33 weeks, and disability due to CP independent of congenital causes or factors during the neonatal period or later, were included in the present study. Causations of CP associated with (cases) and without (controls) umbilical cord complications in each case were retrieved from reports of the Operating Organization of the JOCSC. Patients with undeterminable causes of CP were included as controls. Multiple pregnancies and FHR tracing that could not be evaluated (clear tracing missing from the medical records) were excluded from the present study.

### JOCSC

Details of the JOCSC are described in our previous report [4]. The JOCSC was launched in January 2009 to provide prompt no-fault compensation for children diagnosed with CP caused by trauma during labor and delivery and for their respective families. The JOCSC covers more than 99% of delivery institutions throughout Japan. The JOCSC also provides information that could help in the prevention, early resolution of disputes, and improvement in the quality of obstetric healthcare. A review committee reviews the cases for compensation. After patients are declared as eligible to receive compensation by this review committee, their causes of CP are individually analyzed by the Causal Analysis Committee, which comprises obstetricians, pediatricians, midwives, and lawyers. Once collected, these individual cases are analyzed by the Recurrence Prevention Committee.

### FHR analysis

The National Institute of Child Health and Human Development guidelines [8, 9] were applied when interpreting FHR patterns. We categorized the patients into five groups based on the FHR evolution patterns between admission and delivery, as modified by Phelan et al. [7]. The FHR evolution patterns were retrospectively analyzed by four authors (J.H., M.N., T.I., and E.J.) who were blinded to the clinical characteristics. FHR evolution patterns were determined after careful discussions. Cases with complete lack or partial lack of FHR strips (missing FHR tracing), those with different categorizations among investigators, and those with FHR patterns different from the below mentioned five included categories were excluded from the study.

#### Five groups based on FHR evolution patterns

When an abnormal FHR pattern was observed from the initial FHR examination on admission for delivery, the following two groups were defined: (i) The persistent non-reassuring group comprised fetuses with non-reassuring FHR, usually with decreased baseline variability on admission, and this pattern persisted until delivery. (ii) The persistent bradycardia group comprised fetuses with bradycardia or persistent severe decelerations with loss of variability from the beginning of FHR tracing on admission and no recovery until delivery.

In contrast, when a normal FHR pattern was observed on admission, the following three groups were defined: (iii) The Hon’s pattern group comprised fetuses with a normal FHR pattern on admission. Consequently, there were recurrent severe decelerations (especially variable deceleration) with or without increased baseline heart rate and decreased baseline variability. Finally, prolonged deceleration (PD) or bradycardia occurred before delivery. (iv) The reassuring-PD group comprised fetuses with a normal FHR pattern on admission; however, acute severe PD or bradycardia occurred before delivery. (v) The persistent reassuring group comprised fetuses with a normal FHR range during the entire course of delivery. (Table 1, Additional File 1).

**Table 1:** Definition of FHR patterns

**Abnormal FHR pattern on admission**
** (i) Persistent non-reassuring group**
Fetuses with non-reassuring FHRs usually have a decreased baseline variability at admission for delivery, and this pattern persists until delivery
** (ii) Persistent bradycardia**
Fetuses with bradycardia or persistent severe decelerations with loss of variability from the beginning of FHR tracing at admission for delivery and no recovery until delivery
**Normal FHR pattern on admission**
** (iii) Hon Pattern Group**
Fetuses with a normal FHR pattern at admission for delivery. Consequently, there were recurrent severe decelerations (especially variable deceleration) with or without increased baseline heart rate and decreased baseline variability. Finally, prolonged terminal deceleration or bradycardia occurred before delivery
** (iv) Reassuring-prolonged deceleration (PD) group**
Fetuses had a normal FHR pattern at admission for delivery; however, acute severe PD or bradycardia occurred before delivery
** (v) Persistent reassuring group**
Fetuses with a normal FHR range during the entire course

The umbilical cord complications analyzed in the present study included umbilical cord prolapse, marginal/velamentous cord insertion, multiple cord entanglement, a true knot, umbilical cord constriction, hypercoiled cord, hypocoiled cord, and umbilical cord with a single umbilical artery. The frequencies of FHR evolution patterns were compared between cases with CP associated with umbilical cord complications (cases) and cases with other causes (controls).

### Umbilical cord complications

Findings of umbilical cord complications in the present study were based on macroscopic inspection of the medical records and/or microscopic investigation by obstetricians or midwives in each delivery institution. However, these definitions are usually used in each institution with reference to the Glossary of Obstetrics and Gynecology published by the Japan Society of Obstetrics and Gynecology [8]. Umbilical cord complications include umbilical cord prolapse in which the free loop of the normal umbilical cord accidentally prolapses into the vagina after rupture of the membrane and coexistent antenatal umbilical cord complications, which develop abnormally during pregnancy, such as the following conditions:

#### Velamentous and marginal cord insertion

Velamentous cord insertion is an abnormal cord insertion in which the umbilical vessels diverge as they traverse between the amnion and chorion before reaching the placenta. When the umbilical cord insertion is located just at the edge of the placenta without running through the membranous vessels, it is defined as a marginal cord insertion.

#### Multiple umbilical cord entanglement

Umbilical cord entanglement is the condition when one or more loops of the umbilical cord are encircled around any part of the fetus. In the present study, patients with two or more cord entanglements were included.

#### Umbilical cord constriction/narrow cord

Diagnosis of umbilical cord constriction is made by macroscopic evaluation when the umbilical cord has one or more narrow parts, including umbilical ring constriction, constriction in the free loop, and narrow umbilical cord without Wharton’s jelly.

#### True knot

True knot of the umbilical cord is formed when the fetus passes through a loop of the umbilical cord while being active in the uterus.

#### Abnormal umbilical coiling

The umbilical coiling index was calculated by dividing the total number of coils by the length of the cord in centimeters. Hypercoiled and hypocoiled cords after delivery are defined with umbilical coiling indices of ≥ 0.3 coils/cm (> 90% percentile) and < 0.1 coils/cm (< 10% percentile) [10].

#### Single umbilical artery

Diagnosis of a single umbilical artery is made macroscopically or microscopically when one umbilical artery is absent or obstructed.

### Statistical analysis

A two-sided *p*-value of 0.05 was used to define statistical significance. All analyses were conducted using Stata version 13.0 (Stata Corporation, College Station, TX, USA). Continuous variables were reported as mean ± standard deviation and were compared using Student’s t-test or Mann–Whitney *U*-test. Integer variables were reported as medians and ranges and were compared using the Mann–Whitney *U test*. Categorical variables were reported as frequencies and compared using Fisher’s exact test.

## Results

**Fig. 1:**
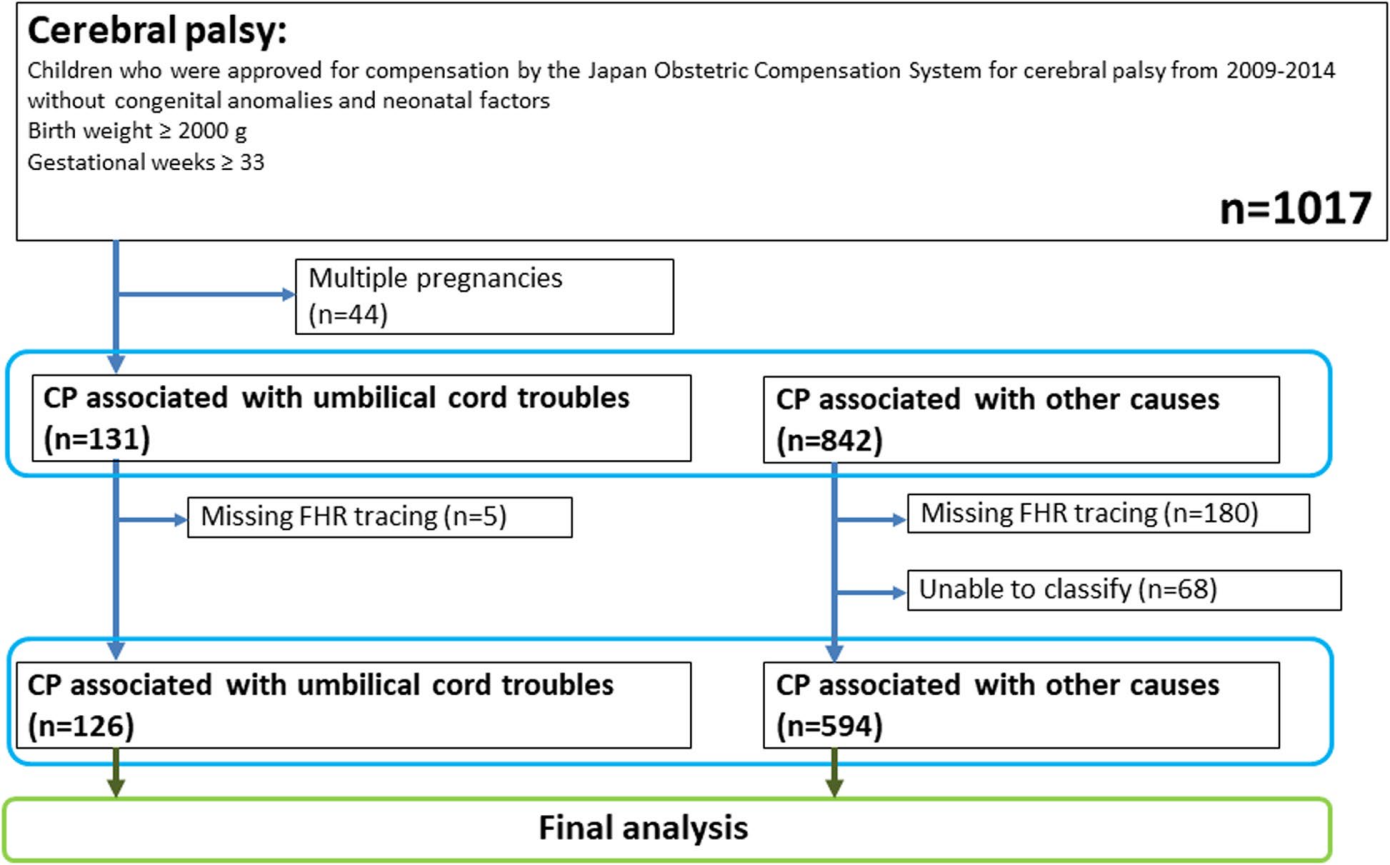
Study flow diagram

The flow diagram of the study is illustrated in Fig. 1. Among 1017 cases of CP from the JOCSC database, after the exclusion of 44 multiple pregnancies, 131 were considered to be associated with umbilical cord complications and 842 were considered to be caused by other reasons. After excluding cases with missing FHR and inability to classify FHR data, 126 cases of CP associated with umbilical cord complications and 594 controls with CP associated with other causes were finally analyzed for FHR evolution patterns.

Characteristics of the patients with CP involving umbilical cord complications and other causes are summarized in Table 2, Additional file 2. Emergency cesarean section rate was significantly higher in patients with CP involving umbilical cord complications than in the controls (*p* < 0.001). Apgar scores in cases of CP involving umbilical cord complications were significantly lower than those in the controls (*p* < 0.001). The use of a cervical ripening balloon was associated with CP involving umbilical cord complications (*p* < 0.001).

**Table 2:** Major obvious obstetric factors considered as association with cerebral palsy

Variables	**Umbilical cord complications** **(Cases: *n* = 131)**		**Other causes** **(Controls: *n* = 842)**		*p* value
***Maternal characteristics***					
Age (yrs)	31.6 ± 5.4		31.2 ± 5.1		0.544
Height (cm)	157.4 ± 5.8		157.6 ± 5.6		0.761
Weight at beginning of pregnancy (kg)	52.5 ± 7.8		53.6 ± 9.6		0.186
BMI (kg/m^2^)	21.2 ± 2.9		21.6 ± 3.8		0.210
Weight at delivery (kg)	62.6 ± 8.2		63.3 ± 9.3		0.392
Weight gain (kg)	10.2 ± 3.9		9.9 ± 4.0		0.380
Parity (median, range)	0 (0–4)		0 (0–5)		0.621
In vitro fertilization	4.6%	(6)	3.4%	(29)	0.516
***Delivery characteristics***					
Premature rupture of the membranes (% (*n*))	23.7%	(31)	23.2%	(195)	0.899
Use of a cervical ripening balloon	**16.8%**	**(22)**	**5.9%**	**(50)**	** < 0.001**
Augmentation (% (*n*))	35.1%	(46)	29.3%	(247)	0.180
Uterine fundal pressure (% (*n*))	20.6%	(27)	17.9%	(151)	0.461
*Mode*					
Normal spontaneous (% (*n*))	**18.3%**	**(24)**	**37.6%**	**(317)**	** < 0.001**
Instrumental (% (*n*))	16.8%	(22)	14.3%	(120)	0.443
Elective CS (% (*n*))	**0.0%**	**(0)**	**4.2%**	**(35)**	**0.017**
Emergency CS (% (*n*))	**61.1%**	**(80)**	**43.9%**	**(370)**	** < 0.001**
*Delivery at*					
Hospital (% (*n*))	56.4%	(74)	62.6%	(527)	0.181
Clinic (% (*n*))	38.9%	(51)	36.6%	(308)	0.604
Midwifery home (% (*n*))	0.8%	(1)	0.8%	(7)	1.000
*Maternal transport after onset of labor* (% (*n*))	6.9%	(9)	10.0%	(84)	0.261
***Neonatal outcomes***					
Gestational weeks	38.6 ± 1.9		38.3 ± 3.1		0.790
Birth weight (g)	2861 ± 449		2858 ± 448		0.950
Birth weight (SD)	-0.31 ± 0.99		-0.17 ± 1.03		0.144
Male (% (*n*))	54.8%	(69)	56.3%	(474)	0.437
Apgar score at 1 min (median, range)	**1 (0–10)**		**3 (0–10)**		** < 0.001**
< 7	**93.1%**	**(122)**	**67.3%**	**(567)**	** < 0.001**
< 4	**76.3%**	**(100)**	**53.4%**	**(450)**	** < 0.001**
Apgar score at 5 min (median, range)	**3 (0–10)**		**5 (0–10)**		** < 0.001**
< 7	**79.4%**	**(104)**	**56.1%**	**(472)**	** < 0.001**
< 4	**48.9%**	**(64)**	**31.6%**	**(266)**	** < 0.001**
Umbilical artery pH (mean, SD)	7.034 ± 0.228		7.053 ± 0.280		0.534
< 7.2	42.7%	(56)	38.0%	(320)	0.300
< 7.0	26.7%	(35)	27.1%	(228)	0.931

Data indicates mean ± standard deviation, median (range), or frequency (*n*)

*BMI* body mass index, *CS* cesarean section, *SD* standard deviation

**Fig. 2:**
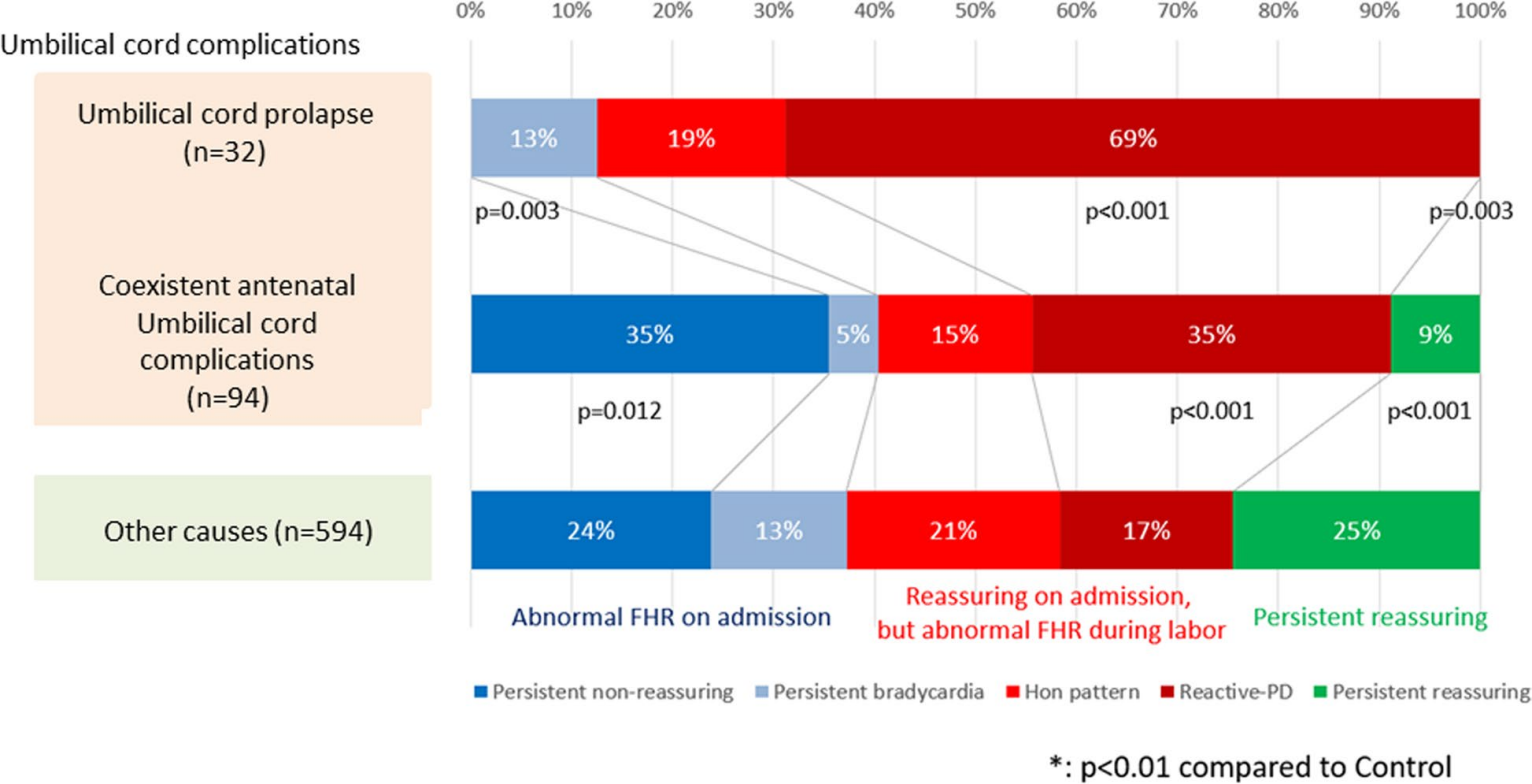
Incidence of abnormal fetal heart rate evolution patterns in cases with cord prolapse, coexistent antenatal umbilical cord complications and control

The incidence of abnormal FHR evolution patterns in cases with cord prolapse, coexistent antenatal umbilical cord complications, and controls is depicted in Fig. 2. Of these, 32 had umbilical cord prolapse, and 94 had other coexistent antenatal umbilical cord complications. Abnormal FHR patterns had already been identified on admission (persistent non-reassuring and bradycardia) in 13% of cases with umbilical cord prolapse, 40% (35% + 5%) of cases with antenatal cord complications, and 37% (24% + 13%) of the controls. Compared with the control, the persistent non-reassuring pattern was more frequent in cases with antenatal cord complications (*p* = 0.012) and less frequent in cases with umbilical cord prolapse (*p* = 0.003). Reassuring FHR patterns were observed on admission, but abnormal FHR patterns during labor were more frequently observed in cases with cord complications than in controls. Reassuring-PD was significantly identified in 69% of cases with umbilical cord prolapse and 35% of cases with antenatal cord complications, compared to 17% of controls (*p* < 0.001). Continuation of reassuring FHR patterns until delivery was significantly less in cases with umbilical cord complications than in the control group.

The timing and type of abnormal FHR evolution patterns stratified according to umbilical cord complications are summarized in Table 3. Abnormal FHR on admission was identified in various types of umbilical cord complications, especially hypercoiled cord (65%) and velamentous cord insertion (50%). In contrast, abnormal FHR patterns were likely to be observed during the first stage of labor in all types of umbilical cord complications, but abnormal FHR patterns during the second stage of labor were identified in 18% of multiple cord entanglements. During the first stage of labor, reassuring-PD was identified in cases of umbilical cord prolapse, cord constriction, hypocoiled cord, and cord with a single umbilical artery.

**Table 3:** Timing and type of abnormal fetal heart rate evolution patterns stratified by umbilical cord complications

	**Abnormal FHR on admission**			**Reassuring on admission**				**Persistent reassuring**
	**Total**	P-Brady	P-NR	**1st stage of labor**			**2nd stage of labor**	
				**Total**	Hon	R-PD		
**Umbilical cord prolapse (*n* = 32)**	**13% (4)**	100% (4)	0% (0)	**84% (27)**	22% (6)	78% (21)	**3% (1)**	**3% (1)**
**Umbilical cord complication coexisting antenatally**								
**Marginal insertion (*n* = 33)**	**48% (16)**	63% (10)	38% (6)	**42% (14)**	50% (7)	50% (7)	**3% (1)**	**6% (2)**
**Velamentous insertion (*n* = 10)**	**50% (5)**	0% (0)	100% (5)	**30% (3)**	100% (3)	0% (0)	**0% (0)**	**20% (2)**
**Multiple cord Entanglement (*n* = 28)**	**29% (8)**	38% (3)	63% (5)	**36% (10)**	70% (7)	30% (3)	**18% (5)**	**18% (5)**
**True knot (*n* = 5)**	**40% (2)**	0% (0)	100% (2)	**40% (2)**	50% (1)	50% (1)	**0% (0)**	**20% (1)**
**Cord constriction (*n* = 18)**	**44% (8)**	38% (3)	63% (5)	**50% (9)**	33% (3)	67% (6)	**0% (0)**	**6% (1)**
**Hyper-coiled cord (*n* = 20)**	**65% (13)**	31% (4)	31% (9)	**30% (6)**	83% (5)	17% (1)	**5% (1)**	**0% (0)**
**Hypo-coiled cord (*n* = 3)**	**0% (0)**			**100% (3)**	33% (1)	67% (2)	**0% (0)**	**0% (0)**
**Single Umbilical Artery (*n* = 7)**	**29% (2)**	50% (1)	50% (1)	**57% (4)**	25% (1)	75% (3)	**0% (0)**	**14% (1)**

*P*-Brady, persistent bradycardia pattern, *P-NR* persistent non-reassuring pattern, *Hon* Hon pattern, *R-PD* reassuring-prolonged deceleration pattern

## Discussion

While the FHR evolution pattern in cases with umbilical cord prolapse was characterized as reassuring-PD, FHR in cases with coexistent antenatal umbilical cord complications, such as hypercoiled cord and umbilical cord placental insertion site, were likely to be both persistent non-reassuring patterns on admission and reassuring-PD during the first stage of labor, compared to cases resulting in CP not associated with umbilical cord complications.

Regarding the timing of the hypoxic-ischemic injury, when the demonstration of an initially normal tracing was followed by patterns of hypoxia/ischemia, such as reassuring-PD and Hon’s patterns, it can be considered that CP was due to intrapartum hypoxic-ischemic injury. Reassuring-PD is considered an acute event causing hypoxic-ischemic damage during labor, while Hon’s pattern is considered a relatively sub-acute stress that affects the fetal brain, causing hypoxic-ischemic encephalopathy.

It is reasonable that the reassuring-PD pattern was identified in various types of umbilical cord complications, especially during the first stage of labor, because an abnormal FHR pattern and insufficient umbilical cord blood flow often originate from the vulnerability of the umbilical cord congenitally coexisting with the developing placenta. For example, in fetuses with velamentous cord insertion, abnormal FHR and perinatal complications are caused by a lack of Wharton’s jelly, which results in the compression of weak membranous vessels [11–14]. Hypercoiled cord could be more prone to cord torsion than to compression or stretching, which adversely affects blood flow during uterine contractions [15, 16]. Variable decelerations are more frequent, especially during the first stage of labor, in cases with a morphologically abnormal umbilical cord, such as velamentous cord insertion and hyper-coiled cord, than in cases without an abnormal umbilical cord [12, 13].

FHR monitoring of fetuses with coexistent antenatal umbilical cord complications often results in Hon’s pattern. We believe that the Hon’s pattern might be caused by the compressive and/or non-compressive failure of umbilical blood flow. Partial or progressive compression of the umbilical cord may lead to a Hon’s pattern. On the other hand, when previously normal tracing evolves slowly to severe abnormality through late or variable decelerations, this also suggests a mechanism of hypoxemic/acidemic basis for the injury, which may be related to a non-compressive failure of the umbilical blood flow. Structural umbilical cord abnormalities that develop during earlier gestation are likely to coexist with placental abnormalities [14, 17]. In such cases, insufficient fetoplacental perfusion during the progression of labor can induce abnormal FHR patterns, resulting in Hon’s pattern. In fact, Hon’s pattern was identified in all cases of velamentous insertion, which is often concomitant with abnormal placental growth.

In abnormal FHR evolution pattern on admission, persistent bradycardia was identified in cases with coexistent antenatal umbilical cord complications. According to us, these patients had already suffered acute or sub-acute compressive failure of umbilical blood flow before admission, resulting in bradycardia and traced FHR after admission.

Contrary to acute and sub-acute adverse events, persistent non-reassuring patterns indicate that causal events occurred over days or weeks before labor onset. In the present study, persistent non-reassuring patterns, such as absent or minimal baseline variability and bradycardia from the beginning of FHR tracing on admission, even before the onset of labor, were present in various types of coexistent antenatal umbilical cord complications. Without overt incriminating cord complications to explain a pre-existing abnormality of fetal tracing, one cannot be certain of the mechanism of preceding injury other than the notion that the timing was likely prepartum. A previous study demonstrated that umbilical cord abnormalities, which can obstruct umbilical cord blood flow, were significantly increased in neonates with placental fetal thrombocoagulopathy (FTV), who later developed CP [3]. According to a previous study, a potentially obstructive pathological umbilical cord, including decreased Wharton’s jelly, a narrow cord, and a hypercoiled cord, is also more frequent in neonates with FTV (30% vs. 9% in those without FTV) [3]. Our results support those of this previous study.

Concerning preventability, those with preexisting abnormal FHR patterns seem unlikely to be preventable (persistent non-reassuring and bradycardia). In addition, prevention of CP in cases with acute severe prolonged deceleration or continuous bradycardia may also be difficult. In a previous study in Japan that demonstrated the prognosis of infants with umbilical cord prolapse, the interval from the diagnosis of prolapse to delivery was found to be significantly longer in infants with a poor outcome than in infants with intact survival (median, 30 vs. 24 min.) [18]. Unless obstetric facilities throughout Japan can provide immediate cesarean section within 15 min, we will not be able to reduce the number of CP cases. Unfortunately, half of deliveries in Japan are managed in private clinics, which cannot perform emergency cesarean sections. However, we recommend initial FHR tracing and evaluation on admission to detect adverse effects related to the onset of labor or rupture of the membrane.

On the other hand, those with initial normal tracing and obvious later deterioration would be at least potentially preventable, especially those with Hon’s pattern. Previous studies have suggested that suboptimal intrapartum care could offer an obvious preventive opportunity for labor asphyxia, particularly in cases with normal admission FHR patterns [19, 20]. The combination of FHR monitoring with timely screening for maternal, obstetric, and fetal risk factors performed much better as a screening modality than FHR monitoring alone [21]. Furthermore, ultrasound screening for umbilical cord abnormalities, triage of pregnant women according to the risks of emergency cesarean section, and appropriate management of FHR and delivery are recommended [22, 23]. Pregnant women with umbilical cord abnormalities diagnosed by antenatal ultrasound should be placed under the continuous FHR observation while preparing for emergency cesarean section when contractions begin.

A limitation of the present study is that the assessment of umbilical cord complications might be slightly different among the different institutions because obstetricians or midwives evaluated umbilical cord abnormalities in their respective delivery institutions. Furthermore, since we only analyzed cases of CP in neonates with a birth weight of ≥ 2000 g and gestational age of ≥ 33 weeks as study subjects, we did not analyze the associations among CP, umbilical cord abnormalities, and fetal growth restrictions.

## Conclusion

Our results suggest that umbilical cord complications that develop antenatally, such as hypercoiled cord and abnormal placental umbilical cord insertion, may be associated with CP due to acute hypoxic-ischemic injury as well as sub-acute or chronic adverse events during pregnancy, while umbilical cord prolapse may be characterized by acute hypoxic-ischemic injury during delivery.

## Supplementary Information

**Additional file 1.**

**Additional file 2.**

**Additional file 3.**

## Data Availability

The datasets generated and/or analyzed during the current study are not publicly available. The JOCSC data were obtained with cooperation from birthing facilities and families. Therefore, it is necessary for this organization to handle personal information with great care, and thus, our policy does not provide outside access to these data. An article in the Standard Conditions of the Japan Obstetric Compensation System stipulates that access to the personal information of children who develop cerebral palsy and their families, as well as of the birthing facilities, is strictly limited to the staff of the JCQHC.

## Abbreviations

CP: Cerebral palsy
FHR: Fetal heart rate
JOCSC: Japan Obstetric Compensation System for Cerebral Palsy
FTV: Fetal thrombocoagulopathy
PD: Prolonged deceleration
P-Brady: Persistent bradycardia
P-NR: Persistent non-reassuring
R-PD: Reassuring-prolonged deceleration
