# Supplementary material for: Fetal heart rate evolution patterns in cerebral palsy associated with umbilical cord complications: a nationwide study
Source: BMC Pregnancy Childbirth. 2022 Mar 3;22:177. doi: 10.1186/s12884-022-04508-2 (PMC8896380; doi:10.1186/s12884-022-04508-2)
Supplement: Supplementary file 1 — Additional file 1. [file 12884_2022_4508_MOESM1_ESM.pptx]

## Slide 1
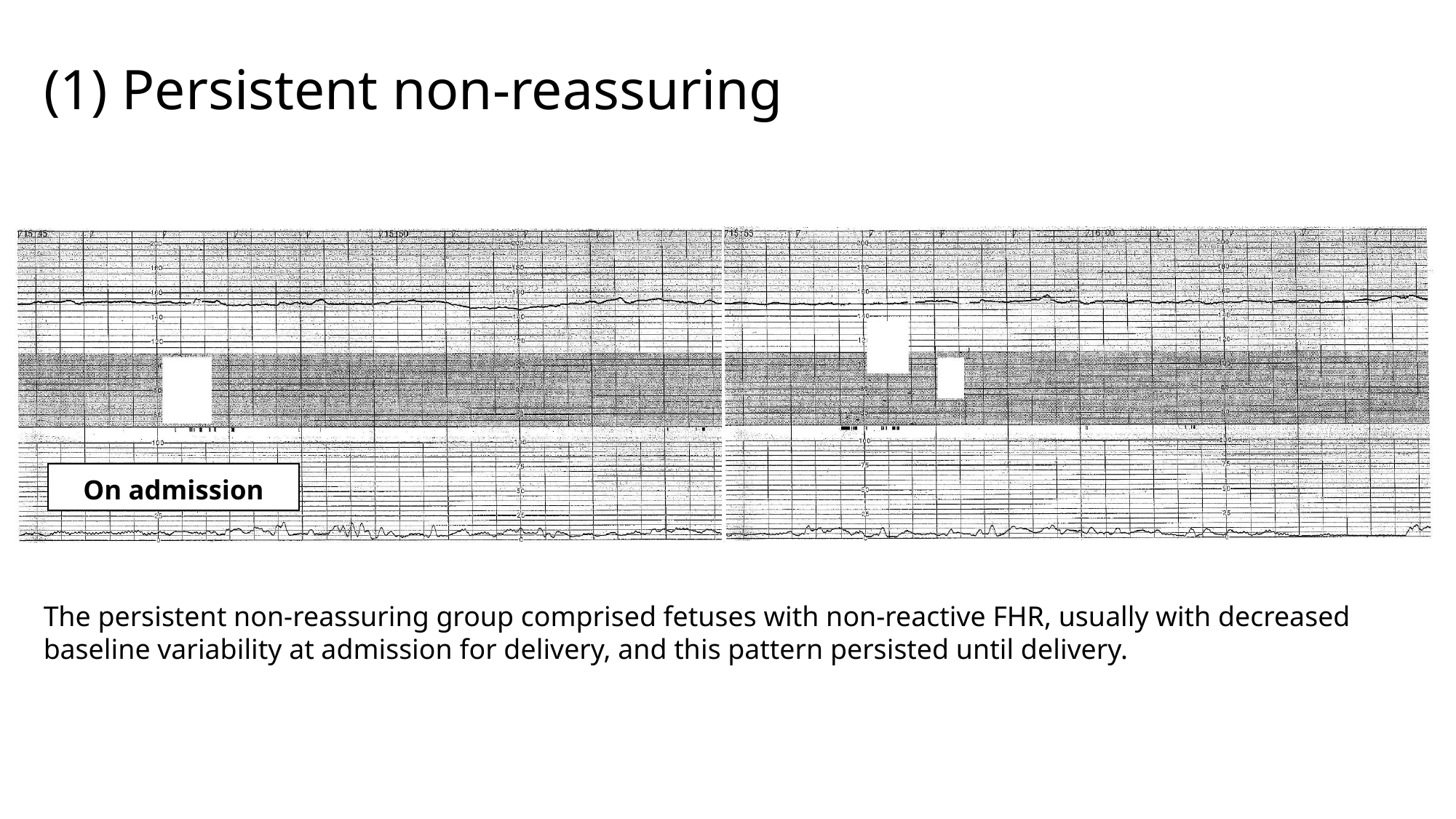

# (1) Persistent non-reassuring
On admission
The persistent non-reassuring group comprised fetuses with non-reactive FHR, usually with decreased baseline variability at admission for delivery, and this pattern persisted until delivery.

## Slide 2
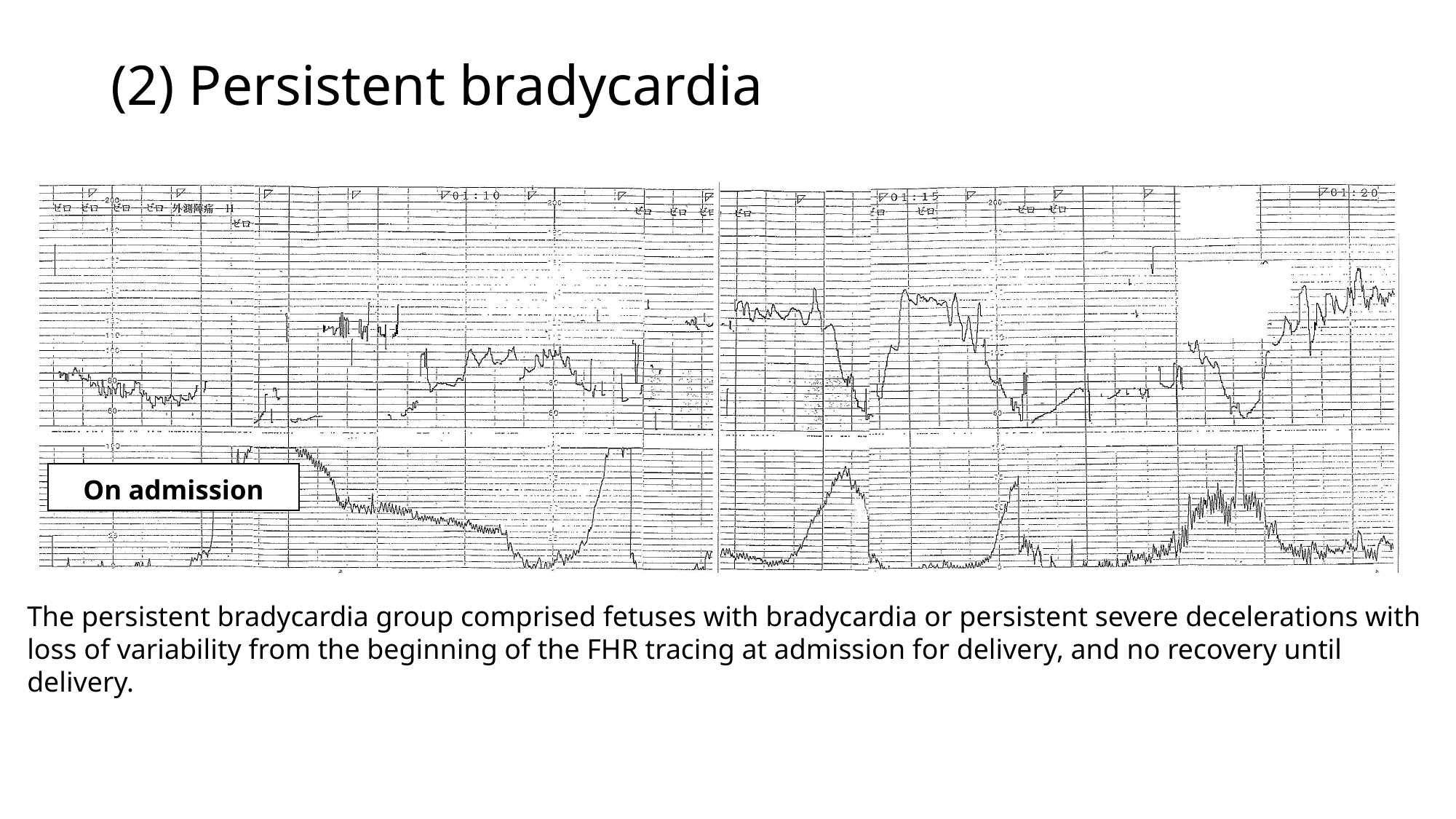

# (2) Persistent bradycardia
On admission
The persistent bradycardia group comprised fetuses with bradycardia or persistent severe decelerations with loss of variability from the beginning of the FHR tracing at admission for delivery, and no recovery until delivery.

## Slide 3
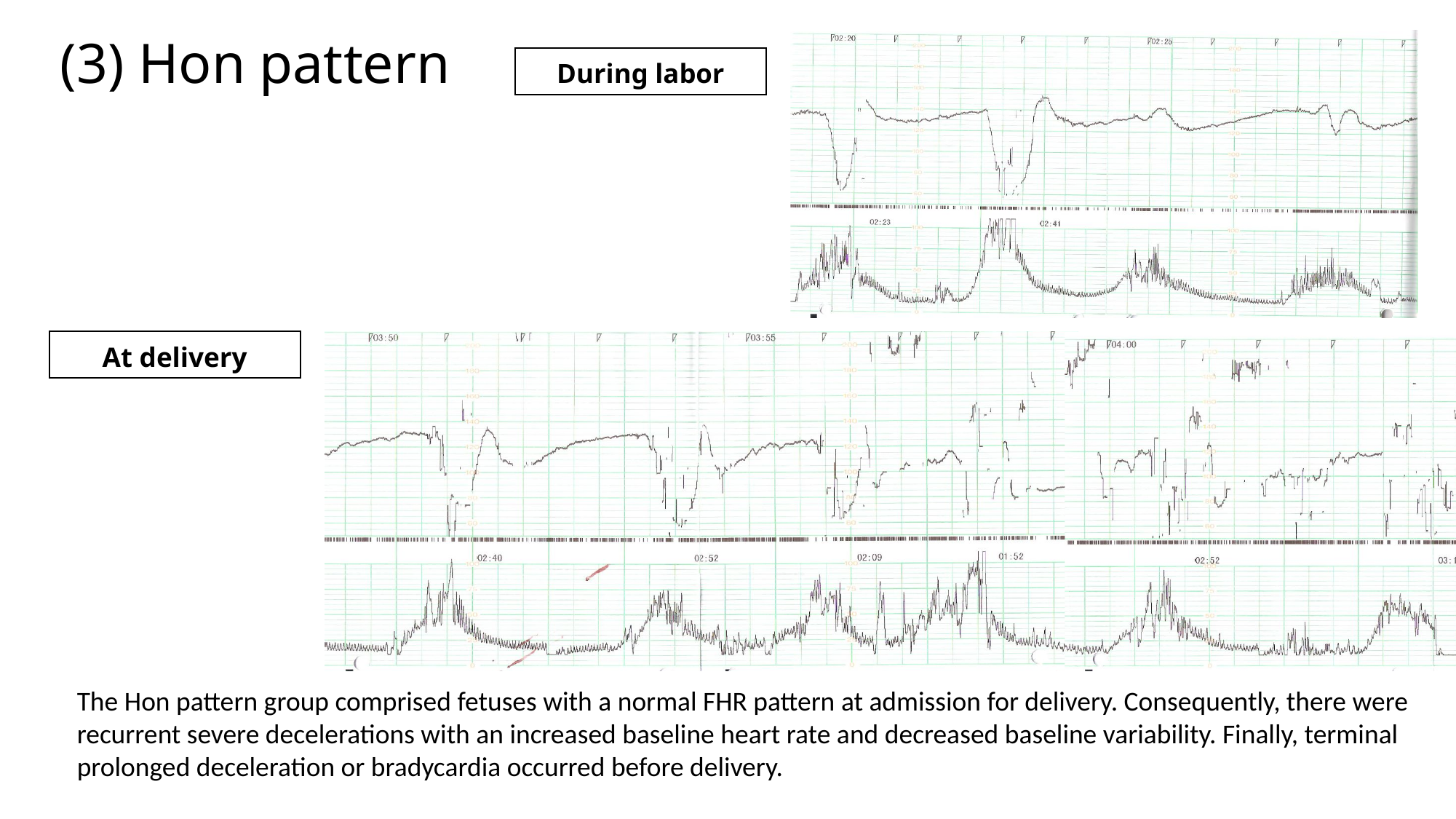

# (3) Hon pattern
During labor
At delivery
The Hon pattern group comprised fetuses with a normal FHR pattern at admission for delivery. Consequently, there were recurrent severe decelerations with an increased baseline heart rate and decreased baseline variability. Finally, terminal prolonged deceleration or bradycardia occurred before delivery.

## Slide 4
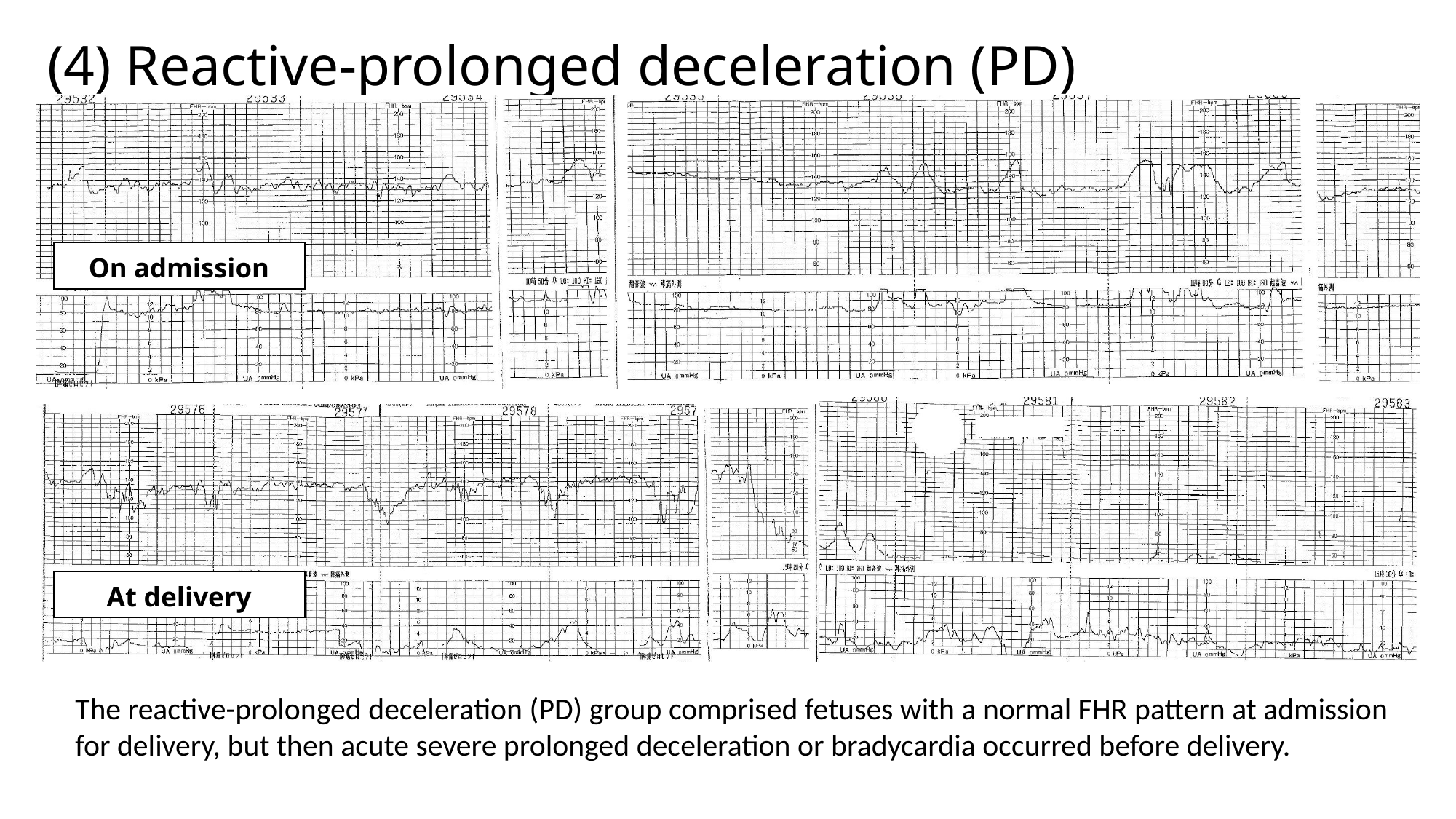

# (4) Reactive-prolonged deceleration (PD)
On admission
At delivery
The reactive-prolonged deceleration (PD) group comprised fetuses with a normal FHR pattern at admission for delivery, but then acute severe prolonged deceleration or bradycardia occurred before delivery.

## Slide 5
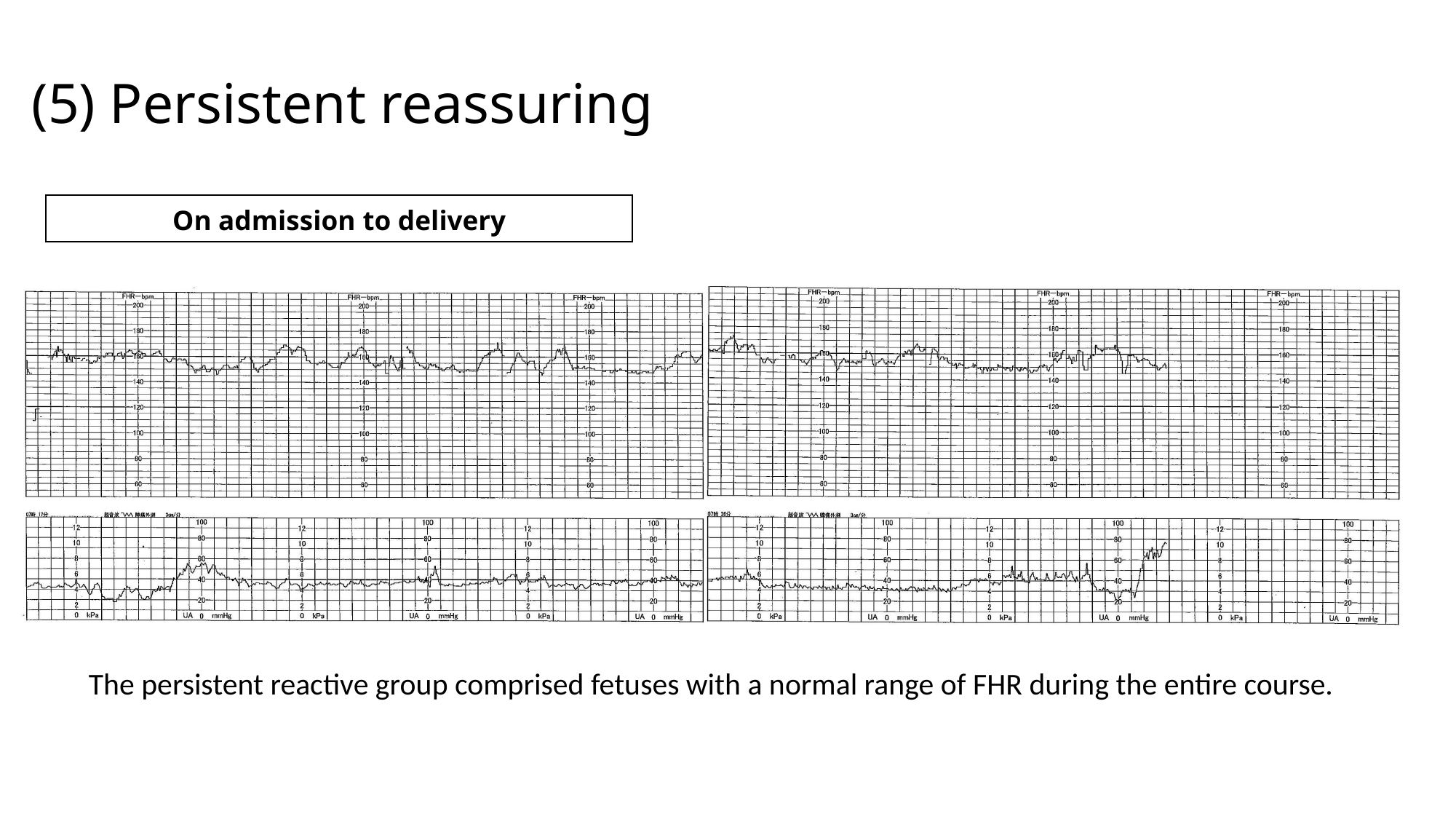

# (5) Persistent reassuring
On admission to delivery
The persistent reactive group comprised fetuses with a normal range of FHR during the entire course.
